# Insufficient Humidification of Respiratory Gases in Patients Who Are Undergoing Therapeutic Hypothermia at a Paediatric and Adult Intensive Care Unit

**DOI:** 10.1155/2017/8349874

**Published:** 2017-04-20

**Authors:** Yukari Tanaka, Sachiko Iwata, Masahiro Kinoshita, Kennosuke Tsuda, Shoichiro Tanaka, Naoko Hara, Ryota Shindou, Eimei Harada, Ryouji Kijima, Osamu Yamaga, Hitoe Ohkuma, Kazuo Ushijima, Teruo Sakamoto, Yushiro Yamashita, Osuke Iwata

**Affiliations:** ^1^Department of Paediatrics and Child Health, Kurume University School of Medicine, Fukuoka, Japan; ^2^Department of Clinical Engineering, Kurume University Hospital, Fukuoka, Japan; ^3^Department of Anaesthesiology, Kurume University School of Medicine, Fukuoka, Japan; ^4^Advanced Emergency Medical Service Centre, Kurume University Hospital, Fukuoka, Japan

## Abstract

For cooled newborn infants, humidifier settings for normothermic condition provide excessive gas humidity because absolute humidity at saturation is temperature-dependent. To assess humidification of respiratory gases in patients who underwent moderate therapeutic hypothermia at a paediatric/adult intensive care unit, 6 patients were studied over 9 times. Three humidifier settings, 37-default (chamber-outlet, 37°C; Y-piece, 40°C), 33.5-theoretical (chamber-outlet, 33.5°C; Y-piece, 36.5°C), and 33.5-adjusted (optimised setting to achieve saturated vapour at 33.5°C using feedback from a thermohygrometer), were tested. Y-piece gas temperature/humidity and the incidence of high (>40.6 mg/L) and low (<32.9 mg/L) humidity relative to the target level (36.6 mg/L) were assessed. Y-piece gas humidity was 32.0 (26.8–37.3), 22.7 (16.9–28.6), and 36.9 (35.5–38.3) mg/L {mean (95% confidence interval)} for 37-default setting, 33.5-theoretical setting, and 33.5-adjusted setting, respectively. High humidity was observed in 1 patient with 37-default setting, whereas low humidity was seen in 5 patients with 37-default setting and 8 patients with 33.5-theoretical setting. With 33.5-adjusted setting, inadequate Y-piece humidity was not observed. Potential risks of the default humidifier setting for insufficient respiratory gas humidification were highlighted in patients cooled at a paediatric/adult intensive care unit. Y-piece gas conditions can be controlled to the theoretically optimal level by adjusting the setting guided by Y-piece gas temperature/humidity.

## 1. Introduction

A number of clinical studies investigated the neuroprotective effect of therapeutic hypothermia to 32–34°C in a range of pathological conditions [1–5]. However, in part because of serious adverse events occurring during cooling, such as septicaemia and ventilator-associated pneumonia, it has been difficult to demonstrate the superiority of moderate hypothermia to conventional normothermic care or targeted temperature management to 36°C except for hypoxic-ischaemic encephalopathy in newborn infants [3–6]. An improved regimen, which reduces systemic adverse events during cooling, may significantly improve the outcome of patients who undergo therapeutic hypothermia at paediatric and adult intensive care units (ICUs).

For mechanically ventilated patients, respiratory gases are humidified close to saturated vapour at 37°C (43.9 mg/L), regardless of body temperature [7]. However, under hypothermic conditions, absolute humidity at saturation decreases in a temperature-dependent manner. Therefore, the default humidifier setting for normothermic condition may provide excessive humidity and condensation in hypothermic patients [8–10]. Indeed, our previous study demonstrated that the default humidifier setting provided excessive gas heating and humidity in patients cooled at neonatal intensive care units [11]. Excessive humidification might be associated with the incidence of adverse events during cooling. However, the incidence of inadequate humidification of respiratory gases and its association with clinical adverse events remains unknown in patients cooled at paediatric and adult ICUs.

The aim of this study was to examine humidification of respiratory gases at the Y-piece of the ventilator circuit in patients of paediatric and adult ICUs who are undergoing therapeutic hypothermia.

## 2. Materials and Methods

This study was conducted under the approval of the Ethics Committee of Kurume University School of Medicine with informed consent from substitute decision-makers of the patients.

### 2.1. Study Population

Between July 2011 and November 2014, 3 children and 3 adults who underwent moderate hypothermia to 33-34°C at a tertiary emergency medical centre in Kurume, Fukuoka, Japan, were studied 9 times over different days (Table 1).

### 2.2. Ventilators, Circuits, and Humidifiers

**Figure 1:**
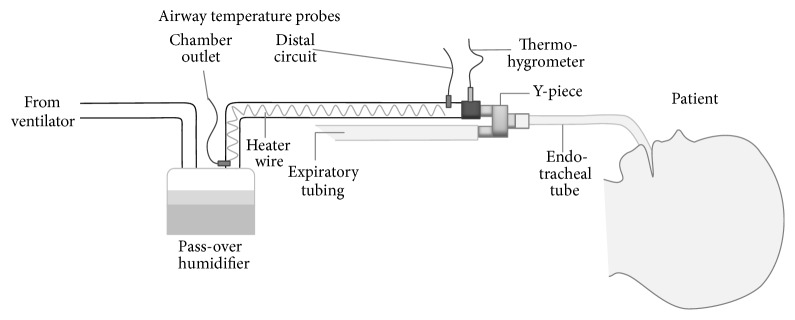
Configuration of the ventilator circuit. A thermohygrometer was inserted between the distal end of the heated inspiratory circuit and the Y-piece. Nonheated extension tubes were not used in the current study cohort.

Three ventilators (the 840, Covidien, Minneapolis, MN; VELA, CareFusion, San Diego, CA; and Evita XL, Dräger, Lübeck, Germany) were used according to the availability and the patient's condition. For these ventilators, oxygen and synthetic air are delivered from the central gas supply except for VELA, which compresses ambient air using its turbine. We used a pass-over humidifier (MR 730; Fisher and Paykel, Auckland, New Zealand) with ventilator-fixed circuits of either Dar (Covidien, for 840) or Evaqua 2 (Covidien, for VELA or Evita XL) (Figure 1).

### 2.3. Data Collection

Data collection from the same patient was repeated up to twice in total with an interval of at least 24 h only after major changes in ventilator settings. Gases were assessed using a clinical, main-stream capacitive thermohygrometer (Moiscope, Siynet, Tokyo, Japan; response time, 3 s within the range of 40 to 100%; accuracy, ±0.5°C for temperature and ±3.5% for relative humidity) [12, 13] placed between the distal end of the inspiratory circuit and the Y-piece (“Y-piece” temperature and humidity) (Figure 1). Although this thermohygrometer is designed for continuous monitoring, we took out the sensor from the circuit to dry it up when studies persisted longer than 10 minutes, or when condensation was observed within the circuit adjacent to the sensor. Temperature values at the humidifying chamber outlet (“chamber outlet” temperature) were provided by the humidifier. Three humidifier settings were serially assessed as follows: (i) 37-default setting (default humidifier setting for normothermic patients; chamber outlet, 37°C; Y-piece, 40°C), (ii) 33.5-theoretical setting (theoretically adjusted humidifier setting for moderate hypothermia by lowering the target chamber outlet temperature and Y-piece temperature to 33.5°C and 36.5°C, resp.), and (iii) 33.5-adjusted setting (humidifier settings adjusted to deliver absolute humidity at saturation at 33.5°C, i.e., a temperature of 36.5°C ± 0.5°C and humidity of 36.6 ± 0.5 mg/L or the closest values at the Y-piece). After the temperature (chamber outlet and Y-piece) and humidity (Y-piece) had become stable with each humidifier setting over 10 minutes, these variables were recorded three times every 3 minutes. The ambient temperature and humidity were also recorded at the beginning of data collection using a capacitive thermohygrometer (605-H1, Testo, Lenzkirch, Germany; accuracy, ±0.5°C for temperature and ±3% for relative humidity). Patients' clinical backgrounds, treatments, adverse events, and short-term outcomes were obtained from the clinical records.

### 2.4. Data Analysis

Values are presented as mean (95% confidence interval) unless otherwise stated. Because of the lack of established thresholds for inappropriate Y-piece gas temperature and humidity during cooling, high and low temperature/humidity was defined based on clinical recommendations for normothermic patients that Y-piece temperature should be controlled to ±2°C from the target level, with its humidity >75% of saturated vapour at body temperature [7]. Thus, the incidence of high temperature >38.5°C (i.e., 36.5 plus 2.0), low temperature <34.5°C (i.e., 36.5 minus 2.0), high humidity >40.6 mg/L {saturated vapour at 35.5°C (i.e., 33.5 plus 2.0)}, low humidity <32.9 mg/L {saturated vapour at 31.5°C (i.e., 33.5 minus 2.0)}, and extremely low humidity <27.4 mg/L {75% of saturated vapour at 33.5°C} was assessed for each humidifier setting.

## 3. Results

Six patients of 28.0 (0.5 to 61.8) {median (range)} years old were studied nine times over different days. Three children used tracheal tubes of 4–5.5 mm in diameter, whereas three adults were intubated with tracheal tubes of 7–9 mm in diameter. Seven and two studies were performed in patients who were ventilated using patient-triggered ventilation and bilevel ventilation, respectively. A turbine air ventilator was used in an adult patient with the fraction of inspiratory oxygen of 0.21 when ambient temperature and humidity were 25.9°C and 36.6%, respectively (patient 6).

### 3.1. Adverse Events

A 5-month-old infant (patient 1) developed pneumothorax and ventilator-associated pneumonia during cooling and required chest drainage and intravenous antibiotics (Tables 1 and 2). Another 61-year-old patient (patient 6) developed ventilator-associated pneumonia during cooling, which was successfully treated using intravenous antibiotics. Tracheal tube obstruction was observed in a 5-year-old child (patient 3) 16 h after the commencement of cooling. Y-piece gas temperature and humidity were assessed shortly after reintubation and were 39.9°C and 28.9 mg/L, respectively, with the 37-default setting.

### 3.2. Setting of 37-Default

Y-piece gas temperature and humidity were 39.1 (38.5–39.8)°C and 32.0 (26.8–37.3) mg/L, respectively. High temperature, high humidity, low humidity, and extremely low humidity were observed in 7, 1, 5, and 2 studies, respectively (Table 2).

### 3.3. Setting of 33.5-Theoretical

Y-piece gas temperature and humidity were 36.4 (36.1–36.7)°C and 22.7 (16.9–28.6) mg/L, respectively. Low humidity and extremely low humidity were observed in 8 and 6 studies, respectively.

### 3.4. Setting of 33.5-Adjusted

Humidifier settings for the chamber outlet and the Y-piece temperature were 37.9 (36.6–39.2)°C and 37.5 (37.0–38.0)°C, respectively (Table 2). Y-piece gas temperature and humidity were 37.5 (37.1–38.0)°C and 36.9 (35.5–38.3) mg/L, respectively. High/low Y-piece temperature and humidity were not observed except for an infant (patient 1) whose Y-piece temperature was 38.6°C.

## 4. Discussion

Use of the default humidifier setting for normothermic conditions in patients cooled at paediatric and adult ICU resulted in higher temperature and lower humidity of Y-piece gases compared with the target levels for hypothermic conditions. This finding contrasted with our previous study in newborn infants, where both Y-piece temperature and humidity were higher than the target levels. Such a trend towards insufficient humidification in the current ICU cohort was exacerbated when the theoretically adjusted humidifier setting for body temperature was used and not observed when the humidifier setting was adjusted according to the Y-piece gas monitoring.

### 4.1. Temperature and Humidity of Respiratory Gases during Therapeutic Hypothermia

For mechanically ventilated patients, respiratory gases are prepared till they become saturated vapour at 37°C (43.9 mg/L) or at least 75% of this level (32.9 mg/L) [7]. Even during therapeutic hypothermia, where absolute humidity at saturation considerably reduces, the default humidifier setting for the normothermic condition is currently recommended. We initially anticipated that the use of this setting in cooled patients may lead to excessive humidification and condensation, which may theoretically cause adverse events, such as mucosal dysfunction and airway oedema [9, 14]. Indeed, our previous study in cooled newborn infants highlighted the common incidence of excessive Y-piece gas humidification when this default humidifier setting for normothermia was used [11]. In the current study, with the 37-default setting, Y-piece gas temperature was higher and humidity was lower than the target levels. Subsequently, with the 37-default setting, Y-piece gas humidity was lower for the current ICU cohort by 7.2 mg/L in average than the level observed in newborn infants despite similar temperature levels [11]. Incidence of systemic complications, such as endotracheal tube obstruction, atelectasis, and ventilator-associated pneumonia, might be investigated in patients, who undergo therapeutic hypothermia at paediatric and adult ICUs, in relation to insufficient humidification of respiratory gases.

### 4.2. Mechanism of Insufficient Humidification in Cooled Children and Adults

The exact mechanism of insufficient humidification in the current ICU cohort remains unknown. Current humidifying systems generally provide only modest humidity as opposed to the ideal level [15, 16]. In addition, insufficient humidity of gases has been reported in association with elevated gas temperature at the inlet of the humidifying chamber [17, 18]. When respiratory gases are already heated by the ventilator and are close to 37°C, the heating power of the humidifier is turned off, where only the temperature, but not the humidity, is prepared for the target level. This is consistent to the current observation of insufficient Y-piece gas humidity despite its paradoxically higher temperature, as well as the exacerbation of insufficient humidification with the 33.5-theoretical setting. However, considering that these mechanisms are likely to affect newborn, child, and adult patients equally, contrasting observations of the Y-piece gas humidity between these cohorts would be attributed to the difference in the detailed ventilator settings, environmental factors, and the patient's clinical variables. For example, compared to newborn infants, respiratory care for child and adult patients would be characterised by the use of balloon-cuffed tracheal tubes of relatively larger diameters, and relatively greater constant flow of the ventilator. Turbine air ventilators, such as VELA in our study, are known to provide relatively warmer respiratory gases, and subsequent humidity of respiratory gases may further be affected by the fraction of inspiratory oxygen and ambient temperature/humidity [19, 20]. The impact of these variables on the Y-piece gas humidity needs to be assessed in future studies by incorporating temperature and humidity monitoring at the chamber inlet and outlet in a sufficient number of child and adult patients.

### 4.3. Potential Solutions for Insufficient Humidification of Respiratory Gases

Our previous and current study suggested that the Y-piece gas condition can be significantly altered by ventilator settings and patients' clinical conditions, suggesting the difficulty in predicting and preventing the incidence of inappropriate humidification of respiratory gases, especially in cooled patients. Nevertheless, when the humidifier setting was adjusted by obtaining feedback from Y-piece gas monitoring, high and low humidification was not observed. The humidifier used in the current study (MR 730) has been replaced by its successor model (MR 850), which provides fully automated adjustment of respiratory gases using feedback from energy consumption and gas flow, resulting in relatively more efficient humidification of gases [15, 18]. The use of MR 850 may reduce the incidence of insufficient gas humidification during cooling, however, with simultaneously increased risks for excessive humidification. Given that the temperature setting of MR 850 cannot be manually adjusted, solution for problems might be difficult. Our previous study suggested that a counter-flow humidifier, which passes gases to a water shower [21], provided perfect temperature/humidity control for cooled newborn infants. The benefit of using the counter-flow humidifier needs to be assessed in further large-scale studies.

## 5. Limitations

There currently is a lack of evidence to support the safety and benefit of a particular strategy in preparing respiratory gases during therapeutic hypothermia. Similarly, there is no established threshold to define inappropriate gas temperature and humidity for cooled patients. In our current study, thresholds for inappropriate Y-piece gas temperature and humidity were defined based on the recommendations released by the International Organization for Standardization for normothermic patients [7], which might be different from the ones associated with clinical adverse events. Our study cohort is too small to confirm their risks and problems. For the same reason, we were unable to highlight the risk factors for inappropriate humidification during cooling. These limitations need to be considered when interpreting our findings to clinical practice.

## 6. Conclusions

Humidification of respiratory gases was different from the level, which are estimated from the humidifier setting in patients who were undergoing therapeutic hypothermia at paediatric and adult ICUs. With the default humidifier setting for normothermic patients, insufficient gas humidification was observed, the trend of which was exacerbated with the humidifier setting adjusted for the hypothermic body temperature. This is the first report to warn of the potential risk of inappropriate humidification in child and adult patients who are undergoing therapeutic hypothermia under mechanical ventilation. When the humidifier setting was adjusted with the feedback from the Y-piece gas temperature and humidity, respiratory gases were successfully humidified to the theoretical target level for hypothermic patients. Future studies are needed to confirm the incidence of inappropriate humidification during cooling in ICU patients and to test whether a new regimen with optimal gas preparation reduces ventilator-associated pneumonia and other serious adverse events encountered during therapeutic hypothermia.

## Figures and Tables

**Table 1:** Clinical background variables of the study population.

Variables	Number (%), median (range) or median {95% CI}
Sex (female)	3 (50.0%)
Age (year)	28.0 (0.5–61.8)
Body weight (kg)	30.4 (5.0–66.0)
Cooling indication	
Cardiopulmonary arrest	3 (50.0%)
Acute encephalopathy	2 (33.3%)
Subarachnoid haemorrhage	1 (16.7%)
Ventilator	
840	4 (66.7%)
VELA	1 (16.7%)
Evita XL	1 (16.7%)
Ventilator mode	
Patient-triggered	5 (83.3%)
Bilevel	1 (16.7%)
Constant flow of ventilator (L/min)	4.9 {3.3–6.6}
Mean airway pressure (cmH_2_O)	10.7 {7.9–13.5}
Fraction of inspired oxygen (%)	0.5 {0.3–0.6}
Ambient temperature (°C)	25.8 {24.9–26.8}
Ambient humidity (%)	37.6 {34.8–40.3}
Core body temperature (°C)	34.2 {33.6–34.9}
Size of endotracheal tube (mm)	5.5 (4.0–9.0)
Adverse events	
Tube obstruction	1 (16.7%)
Pneumothorax	1 (16.7%)
Ventilator-associated pneumonia	2 (33.3%)
Septicaemia	0
Death	0
Days on ventilator	43 (1–88)

**Table 2:** Y-piece gas conditions with three humidifier settings.

Identification		37-default setting					33.5-theoretical setting					33.5-adjusted setting				
		Chamber outlet temperature (°C)		Y-piece temperature (°C)		Y-piece humidity (mg/L)	Chamber outlet temperature (°C)		Y-piece temperature (°C)		Y-piece humidity (mg/L)	Chamber outlet temperature (°C)		Y-piece temperature (°C)		Y-piece humidity (mg/L)
Patient	Study	Set	Actual	Set	Actual		Set	Actual	Set	Actual		Set	Actual	Set	Actual	
1	1	37	36.9	40	39.9	25.1	33.5	33.6	36.5	37.0	18.6	40.5	40.5	38.5	38.6	35.6
	2		37.0		40.0	19.0		33.5		36.4	5.7	40.0	39.4	38.0	38.0	36.4
2	1		37.7		37.1	46.3		33.1		36.3	33.2	37.0	36.3	37.0	36.4	40.2
3	1		37.0		39.9	28.9		33.4		36.6	20.6	37.0	36.5	36.5	37.1	36.2
	2		36.7		37.8	34.3		34.0		37.0	25.9	36.5	35.3	36.5	37.1	36.4
4	1		37.0		39.2	28.8		33.9		35.7	17.6	40.0	39.9	38.0	37.8	36.8
5	1		37.1		39.9	29.9		33.0		36.5	19.2	39.0	39.2	38.0	38.0	36.9
6	1		37.2		39.1	38.0		33.5		35.9	31.9	36.0	35.9	38.0	38.0	36.7
	2		37.2		39.1	38.0		33.5		36.0	31.9	35.0	35.0	37.0	36.8	35.0

Mean			37.0		39.1	32.0		33.5		36.4	22.7	37.9	37.9	37.5	37.5	36.9
95% CI	Lower		36.9		38.5	26.8		33.3		36.1	16.9	36.6	36.2	37.0	37.1	35.5
	Upper		37.2		39.8	37.3		33.7		36.7	28.6	39.2	39.0	38.0	38.0	38.3

Three humidifier settings, 37-default (current clinical recommendation; chamber-outlet, 37°C; Y-piece, 40°C), 33.5-theoretical (theoretically adjusted setting for body temperature 33.5°C; chamber-outlet, 33.5°C; Y-piece, 36.5°C), and 33.5-adjusted (optimised setting to achieve saturated vapour at 33.5°C, or 36.6 mg/L, using feedback from a thermohygrometer), were tested. CI: confidence interval. Set: temperature setting for the humidifier. Actual: actual observations of the temperature value.
